# A Narrative Review of the Association Between Healthy Dietary Patterns and Depression

**DOI:** 10.7759/cureus.60920

**Published:** 2024-05-23

**Authors:** Xeni A Apostolakopoulou, Efthimia Petinaki, Andreas N Kapsoritakis, Konstantinos Bonotis

**Affiliations:** 1 Department of Nutrition, University of Thessaly, Larissa, GRC; 2 Department of Clinical and Laboratory Research, University Hospital of Larissa, Larissa, GRC; 3 Department of Gastroenterology, University Hospital of Larissa, Larissa, GRC; 4 Department of Psychiatry, University of Thessaly, Larissa, GRC

**Keywords:** mental health, depression, healthy dietary pattern, vegetarian diet, dash diet, mediterranean diet

## Abstract

The purpose of the present review is the investigation of healthy dietary patterns and diet quality in relation to depression risk. Nutritional psychiatry is to develop scientifically based research that defines the role of nutrition and nutrients in various aspects of mental health. Growing evidence from the field suggests that diet may play an important role in the prevention and/or treatment of depression. In contrast, there is evidence that unhealthy diets may increase the risk of depression. This emerging research suggests that dietary interventions could help prevent depression or be an alternative or adjunctive therapy for depression.

The Mediterranean diet (MedDiet), the Dietary Approaches to Stop Hypertension (DASH) diet, and the vegetarian diet are examined in this review. The electronic databases PubMed, Scopus, and Google Scholar were searched for relevant studies published during the last five years. We found many results that support that healthy eating patterns (high in vegetables, fruits, whole grains, nuts, seeds, and fish, low in processed foods) are related to a reduction in the risk of depression. The most robust findings are related to MedDiet, where we also found several positive results for the DASH diet. Regarding the vegetarian diet, there are inconsistent reports. Furthermore, a consistent finding refers to a lower Dietary Inflammatory Index (DII) as associated with a lower depression risk.

It has been observed that people suffering from depression have poorer nutritional quality, with lower fruit and vegetable intake. This observation may strengthen the argument that nutritional interventions should be incorporated as an important “pillar” in the multifactorial treatment of patients. However, more well-designed studies are needed to establish the relationship between dietary patterns and mental health. In particular, interventional, longitudinal studies could be more enlightening.

## Introduction and background

Depression is the most common mental health disorder [1], affecting more than 300 million people worldwide, according to the World Health Organisation (WHO) [2]. The etiology of depression includes biological, psychological, and environmental factors. These mechanisms can have synergistic action [3]. In particular, we included studies of the three main healthy eating patterns: the Mediterranean diet (MedDiet), the Dietary Approaches to Stop Hypertension (DASH) diet, and the healthy vegetarian diet. The concept of the MedDiet, as it is now understood in nutritional research, was first described by the Seven Countries Study in the 1950s. This was before the advent of globalization and its impact on lifestyle and diet. Research shows that the Mediterranean diet is beneficial for mental and brain health and is one of the classic anti-inflammatory dietary patterns. This dietary pattern is a plant-based diet that includes high seasonal fruit, vegetables, nuts, and seeds, daily consumption of whole grains, two to three times a week of fish, dairy products, spices, herbs in certain amounts, rare, sweet consumption, low red and processed meat consumption, moderate poultry consumption, olive oil, three to four eggs per week, enough water, and alcohol in moderation. It is linked to an environmentally sustainable and healthy reference diet derived from the EAT-Lancet Commission Report and can address health and environmental issues. In Australia, the study found that successful MedDiet interventions had been implemented [4,5]. According to the American dietary guidelines for 2020-2025, healthy Mediterranean and healthy vegetable dietary patterns are examples of healthy dietary patterns, as they are considered variants of healthy American-style dietary patterns. Similarly, the DASH diet model is an example of a healthy diet model with many of the same features as MedDiet. For example, DASH is characterized by high calcium content, low total fat, and low sodium content [6].

Research has shown a link between a MedDiet of fruits, vegetables, fish, pulses, and nuts and a lower risk of depression [7].

The DASH diet recommends eating vegetables, fruits, and whole grains, including fat-free or low-fat dairy products, fish, poultry, beans, nuts, and vegetable oils, limiting foods high in saturated fat, such as fatty meats, full-fat dairy products, and tropical oils such as coconut, palm kernel, and palm oils, limiting sugar-sweetened beverages and sweets. There are studies associated with a lower risk of depression [7].

Another healthy dietary pattern is vegetarian food. Healthy vegetarian diet patterns can be achieved by incorporating plant protein foods.Τhe healthy vegetable diet pattern is high in soy products (especially tofu and other processed soy products), beans, lentils, nuts and seeds, and whole grains. This is a dairy and egg-based model, including lacto-ovo vegetarians. Meat, poultry, and seafood are not included [6,8].

We conducted a literature review of studies published in the last five years that evaluated the association between healthy eating patterns and depression. This updated review aims to compare the most important healthy dietary patterns and diet quality in relation to the risk of depression.

## Review

Method

This narrative review was performed using the PubMed, Scopus, and Google Scholar databases for all articles published between 2018 and 2023 related to the subject under investigation. Research and review articles were selected. The search focused on scientific articles that examined the association between healthy eating patterns and depression risk. The search was conducted using relevant keywords such as Mediterranean diet, DASH diet, vegetarian diet, healthy dietary pattern, depression, and mental health. Inclusion criteria included articles about healthy dietary patterns and their association with depressive symptoms. We only included studies that involved human participants, clinical trials including randomized controlled trials, observational studies, systematic reviews, and meta-analyses, as well as narrative reviews. Exclusion criteria included studies that measured mental health disorders other than depression, studies that did not focus on the role of MedDiet, DASH diet, and vegetarian diet on depression, studies published in a language other than English, research on eating disorders and the relationship between body weight and depression, as well as animal trials. A total of 43 papers were included in the final review: this included 18 observational studies, seven interventional studies, and 18 systematic reviews and meta-analyses.

Results

Association Between Mediterranean Diet and Depression

The majority of relative studies have examined the relationship between the association of MedDiet with depression, which is summarised in Table 1.

**Table 1:** Data summary of clinical trials and observational studies of the association between Mediterranean diet and depression. SMILES trial, Supporting the Modification of Lifestyle in Lowered Emotional States trial; Mod/MedDiet, ‘Modified’ Mediterranean diet; KIDMED, Mediterranean Diet Quality Index; HELFIMED, Healthy Eating for LiFe with a MEDiterranean-style diet; PREDI-DEP, Mediterranean Diet and Recurrence of Depression; PREDIMED-Plus, Prevención con Dieta Mediterránea-Plus; MEDIS, MEDiterranean Islands Study; DASS-21, Depression, Anxiety, and Stress Scale; NESDA, Netherlands Study of Depression and Anxiety; MDS, Mediterranean diet score; SCID, severe combined immunodeficiency.

Study design	Study population/sample	Method	Results	References
Cross-sectional study	36 young Australian men aged (18-25)	Online questionnaire	Participants following a 12-week Mediterranean diet (MedDiet) intervention achieved positive results in depression symptoms	Bayes et al., 2023 [9]
PREDIMED‑Plus trial	2985 participants	17-item energy-reduced MedDiet questionnaire after 6 and 12 months of follow-up	Changes in dietary recommendations can be achieved by people with high perceived self-efficacy for dietary change	Fernandez-Lazaro et al., 2022 [10]
Cross-sectional study	3172 participants (18–55 years)	Food Frequency Questionnaire (FFQ); Hospital Anxiety and Depression Scale (HADS); General Health Questionnaire (GHQ)	An inverse association was found between MedDiet and the likelihood of mental disorders, including depression	Sadeghi et al., 2021 [11]
Randomized controlled trial (HELFIMED)	182 adults with either GP-diagnosed or self-reported depression via the community using	Newspaper advertisements, flyers, television, radio interviews, social media, market research agency; Depression Anxiety Stress Scale (DASS)	Changing to a healthy diet (MedDiet) can improve depressive symptoms	Parletta et al., 2019 [12]
Randomized, single-blinded trial (the PREDI-DEP study)	222 patients	MD Adherence Screener (MEDAS) questionnaire; semiquantitative FFQ	In patients who have recovered from depression, a positive association was observed between a remote nutritional intervention and the adoption of the MedDiet	Cabrera-Suárez et al., 2022 [13]
Cross-sectional study	143 geriatric patients	MedDiet 14-item questionnaire; 15-item Geriatric Depression Scale (GDS); Mini-Mental State Examination (MMSE); Cumulative Illness Rating Scale for Geriatrics (CIRSG-SI)	MedDiet protects geriatric patients with polymorbidity from the development of depression	Vicinanza et al., 2020 [14]
Cohort (SUN)	14908 participants were included in the present statistical analyses	A validated 136-item semi-quantitative food-frequency questionnaire; Mediterranean Diet Score (MDS) was estimated with the score (0–8 points); a healthy lifestyle score (HLS) (0– 10 points)	Healthy lifestyle habits are correlated with a reduced risk of incidental depression	Ruiz-Estigarribia et al., 2019 [15]
MEDIS study	2718 older people	A quantitative questionnaire and standard procedures; a semi-quantitative, validated, reproducible FFQ; MDS International Physical Activity Questionnaire (IPAQ); Geriatric Depression Scale (GDS)	There was a positive association between daily tea consumption, adherence to the Mediterranean diet, and depression in older adults.	Masana et al., 2018 [16]
Cross-sectional study	435 adult women	A semiquantitative FFQ; Depression, Anxiety, and Stress Scale (DASS-21)	Increased adherence to the Paleolithic and MedDiet may be related to a decrease in the risk of mental disorders such as depression	Zamani et al., 2023 [17]
Netherlands Study of Depression and Anxiety	1634 adults	238-item food frequency questionnaire; Composite International Diagnostic Interview (CIDI) version 2.1 30-item; Inventory of Depressive Symptomatology-Self Report (IDS-SR, range 0–84); 21-item Beck Anxiety Inventory (BAI, range 0–63); 15-item Fear Questionnaire (score range 0–120)	The results show a positive correlation between total MDS ratings and depression	Gibson-Smith et al., 2020 [18]
Clinical trial (PREDI-DEP)	250 people per group.	(arm 1) MedDiet supplemented with extra-virgin olive oil; (control) (arm 2) no nutritional intervention; semi-structured clinical interviews: Spanish SCID-I questionnaire; physical activity questionnaire; FFQ MedDiet Adherence Screener (MEDAS) 14-Item; Short Form-36 Quality of Life Questionnaire (SF-36); Beck Depression Inventory	PREDI-DEP study assesses the importance of MedDiet in preventing the recurrence of depression	Sánchez-Villegas et al., 2019 [19]
Cohort study	49261 participants (aged 29-49).	FFQ; Swedish revisions of the International Classification of Diseases codes (ICD-7: 301.1, ICD-8: 296.2, ICD-9: 296B, and ICD-10: F32 and F33)	Greater adherence to the Mediterranean diet in middle age among Swedish women is associated with a lower risk of depression	Yin et al., 2021 [20]
Study	2092 participants	Mini-Mental State Examination (MMSE); Geriatric Depression Scale (GDS); Mediterranean diet score (Med Diet Score) questionnaires	Increased adherence to MedDiet is correlated with better cognitive status and a reduction in depression symptoms	Mantzorou et al., 2021 [21]
Randomized controlled trial (SMILES)	67 participants dietary intervention, n = 33; control, n = 34	Food diaries; the dietary intervention (Mod/MedDiet); dietary guidelines	The Mod/MedDiet could be an important therapeutic approach in the treatment of depression	Opie et al., 2018 [22]
A descriptive, cross-sectional study	100 inpatients	Eating Habits Questionnaire; the Mediterranean Diet Quality Index (KIDMED) questionnaire (16 items)	The findings showed that the adoption of the Mediterranean diet, and in particular the improvement in dietary habits, was associated with rapid resolution of symptoms and acute stabilization in a short-stay inpatient hospital	Gill et al., 2021 [23]
Randomized, double-blind, controlled clinical trial	60 participants (20-59 years old)	DASS-21 questionnaire; Healthy Lifestyle Score (HLS) questionnaire; 148-item food frequency questionnaire (FFQ); International Physical Activity Questionnaire (IPAQ)	The study suggests that following the MedDiet may improve weight and mental well-being (such as depression), although no clinically significant results were observed	Radkah et al., 2023 [24]

In a study by Bayes et al., which included 36 depressive patients, the authors observed positive highlights for the participants were the enjoyment of the taste of the foods, the motivation to continue the diet and the benefit this had on their depressive symptoms [9]. This could help clinicians promote MedDiet. In a recent study, it was suggested that participants who were more committed to changing their diets and who ate a higher-fiber diet could make greater changes to their recommended diet. Patients with several chronic diseases, especially depression, should be specifically adapted to interventions [10]. Promoting healthy eating habits such as the traditional MedDiet, especially, may be effective both in preventing depression and in complementing antidepressant treatment. In a 2121 study, the MedDiet pattern and risk of mental disorders including depression, anxiety, and distress in 3172 participants were analyzed. The authors found a reverse relationship between MedDiet follow-up and mental disorders, including depression. Specifically, they observed that high fruit and vegetable consumption correlated with reduced depressive symptoms, regardless of whether cereal consumption was associated with depression [11]. Another study investigated whether Mediterranean-style fish oil supplements can improve mental health for depression-affected adults. A total of 182 eligible adults aged 18 to 65 were randomly assigned to receive three months of the Mediterranean diet, three months of MedDiet cooking workshops, and six months of fish oil supplements, or to attend three months of social group [12]. The PREDI-DEP (Mediterranean Diet and Recurrence of Depression) study was a two-year multicentre, randomized, trial evaluating the effect of the Mediterranean diet enriched with extra virgin olive oil (EVOO) on the prevention of depression relapse. Participants were divided into two groups, the intervention group which reduced their intake of vegetables and fruit but to a lesser extent than the control group. The nutritional intervention was delivered remotely every three months. The results showed that EVOO consumption enabled the maintenance of healthy food consumption among patients recovering from depression [13]. Adherence to MedDiet has also been positively associated with a reduction in depressive symptoms in geriatric patients with multimorbidity. Ultimately, this may promote healthy aging in this population [14]. A healthy way of life was associated with a lower risk of depression in the SUN (Seguimiento Universidad de Navarra) group. This study included ten simple healthy lifestyle habits, such as the Mediterranean diet, physical activity levels, physiological body mass index, and other lifestyle changes that may be useful for a more comprehensive approach to preventing depression. These findings underscore the importance of promoting a multidimensional healthy lifestyle to prevent depression [15]. Both adherence to MedDiet and daily tea consumption are associated with reduced depressive symptoms [16]. A cross-sectional study among a sample of Iranian women evaluated the association between paleolithic diet and psychological profiles. Reduced saturated fat consumption and increased monounsaturated and polyunsaturated fat consumption (n-3 PUFAs) are emphasized in the Paleolithic and Mediterranean dietary patterns. In particular, increased intake of monounsaturated fatty acids (MUFAs) promotes insulin signals in the brain and maintains the integrity of the brain's dopamine system, thus reducing the risk of depression [17]. An increase in the consumption of non-raised grains, vegetables, and alcohol is associated with a total Mediterranean Diet Score (MDS) and a lower depression/anxiety score [18]. Sánchez-Villegas et al. evaluated the correlation between adherence to MedDiet and the prevention of recurrent depression [19]. Regarding the observational studies, the largest study (49261 women) showed that consumption of food included in MedDiet, especially, supplemented with fish in middle age among Swedish women linked to reduced risk of depression in later life [20].

Another recent study identified that increased adherence to MedDiet has been positively related to a lower degree of depression [21]. Furthermore, a recent study showed that there may be an improvement in nutritional quality in people with major depressive disorder if they receive the appropriate nutritional advice from qualified dietitians. The SMILES (Supporting the Modification of Lifestyle in Lowered Emotional States) trial was the first randomized controlled trial (RCT) to evaluate a dietary intervention to reduce depressive symptoms in people with depression [22].

The results of a descriptive, cross-sectional study demonstrated the prevalence of unhealthy dietary habits in psychiatric inpatients. The results suggest that improved dietary habits such as MedDiet have a faster resolution of symptoms in a short-stay hospital unit. Seventy percent of the study population were suffering from depression; this percentage as well as the average unhealthy habits correlates with the two-way relationship between depression and poor eating habits. The authors mention that they did not correct for cofounders [23].

Another study was conducted in Iran and included 20-59-year-old participants from a psychiatric clinic with depression, stress, or anxiety diagnosed for over a month and had BMIs 18.5 to 40. A reduction in depression, anxiety, stress, and physical measurements was observed after adjusting for confounding variables [24].

The findings of six meta-analyses showed that greater adherence to MedDiet was correlated with a lower risk of depression. Adhering to a healthy diet especially the MedDiet seems to have protection against depression in observational studies. This underlines how important dietary interventions are in preventing depression [25-27]. A comprehensive review evaluated the association between different dietary patterns and depression. Higher adherence to MedDiet, as well as lower Dietary Inflammatory Index (DII) scores, was significantly associated with a lower risk of depression [28]. A meta-analysis by Wu et al. found that healthy diets like the Mediterranean and DASH diets are associated with a lower risk of depression in older adults [29]. Α meta-analysis of sixteen eligible randomized controlled trials observed that nutritional interventions were associated with a low risk of depression. Women with depression who followed diet interventions had positive results [4]. This study also found that adults who followed a healthy eating pattern had lower depressive symptoms and a lower risk of depressive symptoms.

Association Between DASH Diet and Depression

Several studies in the literature have investigated the relationship between the DASH diet and depression which are summarized in Table 2. In another study of patients with metabolic syndrome in which the follow-up period lasted seven years, it was observed that (among other lifestyle interventions) a modified DASH diet was associated with improvement after 24 weeks of follow-up, in terms of quality of life anxiety, depression, and stress [30]. We found two interventional clinical studies: sixty-six diabetic women participated in this RCT. They were randomized into two different diet groups (DASH and control). Thirty-three patients in each group for three months evaluated that the DASH diet improved biochemical markers in the intervention group and had beneficial effects on depression, anxiety, and stress scores, as well as sleep status [31].

**Table 2:** Data summary of clinical trials and observational studies of the association between DASH diet and depression. DASH, Dietary Approaches to Stop Hypertension; MIND, Mediterranean-DASH Intervention for Neurodegenerative Delay; HANDLS, Healthy Aging in Neighborhoods of Diversity Across the Life Span; BMI, Body Mass Index.

Study design	Study population/sample	Method	Results	References
A randomized controlled trial	145 participants	Quality of life (Short-Form 36 Health Survey Questionnaire, SF-36); anxiety/depression (Hospital Anxiety and Depression Scale, HADS); stress (Cohen Perceived Stress Scale, CPSS); mood (Profile of Mood States, POMS); self-efficacy (General Self-Efficacy Scale, GSE); mindfulness (Mindfulness Attention Awareness Scale, MAAS); self-compassion (Self-Compassion Scale, SCS)	Τhe results of this study point to beneficial and clinically relevant effects of fasting and intensified lifestyle modification on quality of life and psychological parameters including depression	Jeitler et al., 2022 [30]
A randomized clinical trial	66 participants	Two diet groups; DASH diet, control diet; 33 patients in each group; Pittsburgh Sleep Quality Index; Depression, Anxiety, Stress Scale-21 items; a questionnaire (sociodemographic information); dietary records; weekly phone calls; serum level of vitamin C	A 12-week DASH diet is associated with lower depressive symptoms in type 2 diabetes patients	Daneshzad et al., 2022 [31]
Cross-sectional study	535 girls aged (12 and 18 years)	A standard questionnaire socioeconomic status (SES); Modifiable Activity Questionnaire (MAQ); a 168-item food frequency questionnaire; DASH scores; Beck Depression Inventory (BDI) a Persian version; Buss-Perry questionnaire for the assessment of depression and aggression	Greater adherence to a DASH diet was associated with a lower risk of depression in adolescent girls	Khayyatzadeh et al., 2018 [32]
An observational prospective cohort study	709 participants (23.3% men, mean age 80.4	A validated food frequency questionnaire for the DASH diet; Mediterranean diet, MIND diet, Western diet; a generalized estimating equation (GEE) model was performed for the longitudinal analysis of depression	Elderly adults with higher DASH and MIND diet scores had reduced depressive symptoms, in contrast to those on a Western diet	Cherian et al., 2021 [33]
HANDLS study	3720 HANDLS participants	Center for Epidemiologic Studies Depression Scale (CES-D); 24 h recalls; DASH diet was determined for each participant	Results of multiple mixed-effects linear regression models indicated an inverse cross-sectional relationship between DASH (mean) and BMI (mean), in women and participants with incomes <125% of poverty	Kuczmarski et al., 2019 [34]
Α population-based cohort study (The Maastricht Study)	3451 participants	9-item Patient Health Questionnaire (PHQ-9); a validated Food Frequency Questionnaire [Dutch Healthy Diet (DHD), Mediterranean Diet, and Dietary Approaches To Stop Hypertension (DASH)]	Adherence to MedDiet, DASH, DHD diet was not associated with depression	Gianfredi et al., 2021 [35]
Α cross-sectional observational study	266 overweight and obese women	depression, anxiety, and stress scale (DASS) score; Pittsburgh Sleep Quality Index (PSQI); Morning–Evening Questionnaire (MEQ); a validated semi-quantitative Food Frequency Questionnaire (FFQ)	There is an important correlation between a DASH diet and MedDiet with mental health	Shiraseb et al., 2023 [36]
Α case-control study	246 eligible adults (123 cases and 123 controls)	Food frequency questionnaire (FFQ) 68-item; Depression, Anxiety Stress Scales (DASS) 21 items; Pittsburgh Sleep Quality Index (PSQI) 19-item; Quality of Life Questionnaire Short Form Health Survey (SF-36)	Following the DASH diet may reduce depression and stress in recovered COVID-19 patients	Khorasanchi et al., 2022 [37]

There was a reverse correlation following the DASH diet with reduced odds of depressive symptoms in adolescent girls and no association between the DASH-type diet and aggression [32]. Some adjustments for cardiovascular disease and physical activity were added, but the results showed no change [33]. This study examined the association between depressive symptoms assessed using the CES-D (Center for Epidemiologic Studies Depression) scale and the quality of the diet assessed using the DASH score and BMI. An inverse relationship between DASH and BMI was observed, mainly in women who agreed with several cross-sectional studies regarding adherence to DASH and its association with lower BMI. However, the HANDLS (Healthy Aging in Neighborhoods of Diversity across the Life Span) study found a negative association between DASH diet quality and depression [34]. An observational study found that The DASH diet and the MIND (Mediterranean-DASH Intervention for Neurodegenerative Delay) diet are linked to a lower rate of depressive symptoms over time, in contrast to the Western diet which, was correlated with higher rates of depressive symptoms over time. In this study, in which the follow-up period lasted seven years, it was observed that higher adherence to the Dutch Health Diet was associated with reduced risk of depression, whereas following the Mediterranean diet and DASH diet was not associated with incidences of clinically relevant depressive symptoms [35]. A cross-sectional observational study investigated the correlation between DASH and Mediterranean diet scores and mental health, sleep, and circadian rhythms. There was also a significant reverse association between adherence to the DASH diet and major depression [36]. In a recent study, it was observed that high adherence to a DASH diet that includes high consumption of vegetables, fruits, legumes, and nuts, and low consumption of meat was correlated with low depression and stress in a recovered patient with COVID-19 [37]. A cross-sectional study conducted to assess adherence to the DASH diet and depression in adolescent girls found that adults who followed a healthy eating pattern had lower depressive symptoms and a lower risk of depressive symptoms. The following dietary patterns were investigated in relation to depressive symptoms, MDS, Alternative Healthy Eating Index (AHEI-2010), and DASH [38].

Association Between Vegetarian Diet and Depression

We found only a few recent observational studies on the vegetarian diet and depression (Table 3). What is observed is that some studies show a higher risk of depression in vegetarians than others that do not observe an association or that vegetarian diets may be beneficial for depression. In the studies analyzed, three studies showed that vegetarian diets were associated with a lower risk of depression [39,40]. Lastly, in a cross-sectional study, no significant association was noted between plant-based diet index (PDI) and healthful PDI (hPDI) scores and obesity while unhealthful PDI (uPDI) values showed an increased risk of obesity. Higher PDI and hPDI scores were associated with a decreased risk of depression. On the contrary, higher uPDI values showed a direct association with depression [17]. For example, a large cohort study in Taiwan (12061 participants) showed that a vegetarian diet was correlated with a lower risk of depressive symptoms. However, more studies found an increased risk of depression in vegetarians [41-44,8], whereas others found no association [45-47], or that a vegetarian diet may be beneficial for depression [48]. Evidence regarding the association of vegetarians and diet with depression is conflicting [49].

**Table 3:** Data summary of observational studies of the association between vegetarian diet and depression. CESD, Centre for Epidemiological Studies of Depression Scale; EQ-5D-3L, EuroQol; GAD2, Generalized Anxiety Disorder Scale; PGQ Scale, Patient Health Questionnaire Scale; NHANES, National Health and Nutrition Examination Surveys; DASS, Depression Anxiety Stress Scale; WEMWBS, Warwick‐Edinburgh Mental Well‐being Scales; PREDIMED-Plus, Prevención con Dieta Mediterránea-Plus; GP, General practitioner.

Study design	Study population/sample	Method	Results	References
Cross-sectional study	435 women	A validated and reliable semi-quantitative food-frequency questionnaire; DASS-21 questionnaire	Unhealthy plant-based foods were associated with an increased risk of depression	Zamani et al., 2020 [17]
Cross-sectional study	496 participants	Depression (CESD-20) 20-item; Dietary Screening Tool (DST) 37 items; International Physical Activity Questionnaire (IPAQ); the Social Connectedness Scale	A high-quality plant-based diet associated with reduced depressive symptoms	Walsh et al., 2023 [39]
A prospective cohort study, the Tzu Chi Vegetarian study (TCVS)	12062 participants	A detailed food frequency questionnaire (FFQ); 57-item Nutrition and Health Survey in Taiwan (NAHSIT)	A reduced likelihood of later depression was observed in a study of Taiwanese vegetarians and non-vegetarians	Shen et al., 2021 [40]
Epidemiological study	9668 adult male	Edinburgh Post Natal Depression Scale (EPDS); Avon Longitudinal Study of Parents and Children (ALSPAC)	The study found that vegetarian men exhibited a higher prevalence of depressive symptoms and nutritional deficiencies in iron or cobalamin, which may be a contributing factor. However, it is important to note that reverse causation cannot be ruled out	Hibbeln et al., 2018 [41]
Cross-sectional and longitudinal analyses	3020 people from Russia. 3038 people from the USA. University students from Germany (1608), China (12744)	BOOM (Bochum Optimism and Mental Health) studies; telephone interviews; Quality of health (EQ-5D-3L); Family affluence scale; (DASS-21) 21-item	Α positive association with vegetarianism, anxiety and depression was found in China while in the US, Russia or Germany no association of vegetarianism with mental health was observed	Lavallee et al., 2019 [42]
Population-based, prospective cohort (Constances)	90380 subjects	(CES-D) scale; FFQ	Several studies have observed a positive association between vegetarian diets and good physical health. However, the exclusion of any food group has been associated with an increased risk of depression. This suggests that there may be an association between depression and the exclusion of any food group, including vegetarian diets	Matta et al., 2018 [43]
Cross‑sectional survey	6578 participants aged 18-90, 70.8% females	Self-reported questionnaire data; GAD-2 scale anxiety; PGQ-2 scale depression	No association between a plant-based diet and depression or anxiety was found	Bègue et al., 2022 [45]
Cross-sectional, population-based data (NHANES)	9584 individuals	US National Health and Nutrition Examination Surveys; Patient Health Questionnaire (PHQ-9)	A vegetarian diet was not found to be associated with PHQ-9 defined depression	Storz et al., 2023 [47]
Cross-sectional study	219 adults aged 18–44	20-item Centre for Epidemiological Studies Depression (CESD); Dietary Screening Tool (DST) 20-item questionnaire	This is the first study in Australian adult vegans and vegetarians that shows a correlation between plant-based diet quality and depression	Lee et al., 2021 [48]
Preliminary cross‐sectional study	57 participants between the ages of 18–40	Nordic Physical Activity Questionnaire; Big‐Five Inventory (BFI); Empathy Quotient 10 (EQ‐10); DASS; Warwick‐Edinburgh Mental Well‐being Scales (WEMWBS); Pittsburgh Sleep Quality Index (PSQI); Dietarian Identity Questionnaire (DIQ); EPIC Food Frequency Questionnaire (FFQ)	These preliminary findings suggest that not only personality traits, but also plant-based diets have been associated with better mental health	Coxon et al., 2023 [49]

Furthermore, one of the three meta-analyses analyzed in this review found no association between a vegetarian diet and depression [46]. On the contrary, in the other two meta-analyses, no significant differences were observed between vegetarians and omnivores, although a subgroup analysis showed a statistically significant higher level of depression in vegetarians under the age of 26 years [44]. Furthermore, other meta-analyses showed that a vegetarian diet significantly increased the risk of depression, however, the results were not robust [8].

Discussion

In our review, a plethora of studies suggest a probable beneficial association between healthy eating patterns and depression, mainly related to MedDiet [7,50]. Interestingly, we show that interventions for depression based on MedDiet and lifestyle interventional trials based on MedDiet show positive results [11,13,14,19,20,22,24]. Specifically, all fifteen studies on the MedDiet dietary pattern confirmed a lower risk of depression associated with its adoption, concluding that the MedDiet has positive effects. Additionally, οut of the eight studies analyzed, six showed a positive association between the adoption of the DASH dietary pattern and depression, while only two showed a negative association. On the other hand, after examining ten studies in total, it was observed that there is a positive association between the adoption of the vegetarian diet and depression in six studies, while four studies showed a negative association. After analyzing all dietary patterns in meta-analyses [25-29], they were found to be consistent with the studies [4,8,44,46]. Moreover, in all meta-analyses, healthy eating patterns and diet, in general, were critical factors for depression.

It is important to mention that the majority of studies that evaluate the association between healthy eating patterns and depression have been observational studies. Particularly, there is a lack of clinical interventional trials on healthy eating patterns (except MedDiet) at least during the last five years. Whereas in cross-sectional studies confounding factors interfere. This probably indicates that this is a relatively new area of research that needs more clinical trials. Hence, the methodology should be longitudinal and interventional. Of particular interest may be the study of a dietary intervention with anti-inflammatory properties, such as the healthy eating patterns we report, as an adjuvant in subpopulations of depressed patients [51].

There appear to be several biological mechanisms that could interfere with the impact of diet on mental health. These factors include inflammation, oxidative stress, insulin resistance, and changes in vascularity. All of these mechanisms can be modified by diet and are associated with the onset of depression [25,7]. Meta-analysis of prospective population-based observational studies (33 articles) indicated that the consumption of pro-inflammatory and Western diets was associated with an increase in depression, while the consumption of fruits and vegetables was associated with a decrease in depression. It has been found that consuming diets that include ultra-processed foods can increase the risk of developing depression. Moreover, depression is also associated with increased levels of proinflammatory cytokines [7]. On the contrary, the biological mechanisms underlying the associations between healthy dietary patterns and depression risk are likely to be due to the biological effects of different dietary components. These foods have antioxidant and anti-inflammatory properties. Several nutrients have been shown to reduce inflammation in the body. In particular, dietary magnesium can reduce plasma C-reactive protein, an acute-phase marker of inflammation. Specifically, magnesium sources include greens, nuts, seeds, legumes, and grains that are widely used in vegetarian diets [46]. The mechanisms by which a pro-inflammatory diet could increase the risk of depressive symptoms [52] may be through pro-inflammatory nutrients activating the innate immune system, which can lead to low-level inflammation and chronic diseases such as cardiovascular and depression. An important correlation between a pro-inflammatory diet (processed foods) and an elevated risk of depression, compared to those with an anti-inflammatory diet (fruits and vegetables).

The dietary index DII has been created to assess a person's overall diet and its effect on inflammation. With regard to this, DII is a scoring algorithm to classify individuals' diets according to their inflammatory potential, based on 45 biomarkers. This has been successfully validated using various inflammatory markers [25,52]. A healthy diet, particularly traditional MedDiet, DASH, vegetarianism, or the absence of pro-inflammatory diets, seems to provide some protection against depression. Ηigher adherence to MedDiet, generally higher fruit and vegetable consumption, and lower DII scores with anti-inflammatory properties were associated with a lower risk of depression [20,28,52,53].

People with depression appear to have higher inflammatory potential dietary intake based on DII scoring [15,27]. A healthy diet also includes the necessary quantities of omega-3 fatty acids, B6, and B12 vitamins, magnesium, zinc, and other essential nutrients that are essential to normal functioning, and the insufficient intake of these nutrients is associated with an increased risk of depression, so deficiencies must be addressed. Especially vegetables and fruits in particular are the main components of these healthy eating patterns and contain many nutrients, including the following vitamins and minerals are present: A, C, K, E, B6, folic acid, copper, magnesium, iron, thiamine, niacin, and choline. Other elements of the Med and DASH diets include whole grains, fish, seafood, zinc, iodine, magnesium, copper, and other nutrients [54,32,55].

Fruit and vegetables rich in antioxidants, especially polyphenols, are the main food of vegetarians, and have a negative relationship with depression. Antioxidants, which are found in high levels in fruits and vegetables, can help protect the brain from oxidative damage [53,7].

In contrast, nutritional deficiencies in plant foods are associated with an increase in depression symptoms [25]. In an epidemiological study, we observed that vegetarians have a lower intake of n-3 PUFAs, vitamin B12, and folic acid and a higher intake of nuts, which may be associated with high levels of omega-6 fatty acids with an increased risk of depression. Associations between elevated homocysteine or low vitamin B12 levels and the risk of major and minor depression have been reported in several observational studies. So, nutritional deficiencies are a possible explanation for these findings [41].

Beyond inflammatory processes, other biological mechanisms described are related to neurophysiological processes. A healthy diet includes fruits and vegetables, which contain folic acid, which is involved in the synthesis of some neurotransmitters such as dopamine, serotonin noradrenaline, and glutamate [25].

A new paradigm has been described, the brain-gut-microbiome axis, to understand the pathophysiology of depression. The brain-gut-microbiome-brain axis is a two-way communication system that allows intestinal microbes to communicate with the brain and vice versa [56,57]. Recent studies show that patients with depression have an altered microbiome composition (dysbiosis). This observation is being investigated in relation to the influence of nutritional factors. They are also rich in fiber, which plays a major role in the proper functioning of the gut microbiota and the gut-brain interface [8]. There are studies focusing on key food components, such as flavonoids(polyphenols) that have a positive effect on the gut microbiome and need to be studied further [58,59]. More studies are needed to strengthen the role of microbiome as an intervening factor in diet-depression link. Independently from the study of associated biological mechanisms, there is much data supporting that healthy dietary patterns, particularly MedDiet, may have a protective role in mental health, especially depression. However, in view of the heterogeneity of the presented studies, further research is needed [28]. It is crucial to highlight the significance of embracing a healthy dietary pattern in its entirety, as described in our review. This is because the ultimate outcome depends on the complete nutritional pattern and not just individual components.

## Conclusions

In recent years, many studies have shown the importance of healthy dietary patterns in the prevention and control of depression. The robust findings concern the Mediterranean diet (MedDiet) where we also found several positive results for the Dietary Approaches to Stop Hypertension (DASH) diet and regarding the vegetarian diet, there are inconsistencies. From a public health perspective, an approach of avoiding pro-inflammatory diets and adopting anti-inflammatory diets, and healthy eating patterns as mentioned in this review, would be feasible. Nutritional psychiatry in the future must aim to prevent and treat depressive symptoms. Finally, more data and intervention studies are needed to support the importance of adopting healthy eating patterns in preventing and controlling depressive symptoms. Studies focusing on patients' adoption of healthy eating patterns and investigating their impact on disease prevention or progression would enrich the existing literature on the relationship between diet and depression. Nutritional psychiatry defines the roles of nutrition and nutrients in mental health and nutritional interventions as adjuvant treatments for depression.
